# Pharmacogenomic Landscape of Ivermectin and Selective Antioxidants: Exploring Gene Interplay in the Context of Long COVID

**DOI:** 10.3390/ijms242015471

**Published:** 2023-10-23

**Authors:** Ying-Fei Yang, Sher Singh

**Affiliations:** 1 Department of Bioenvironmental Systems Engineering, National Taiwan University, Taipei 10617, Taiwan; f00622037@ntu.edu.tw; 2 Department of Life Science, School of Life Science, College of Science, National Taiwan Normal University, Taipei 11677, Taiwan

**Keywords:** long COVID, ivermectin, antioxidant compounds, pharmacogenomics, network pharmacology, disease analyses

## Abstract

COVID-19 pandemic has caused widespread panic and fear among the global population. As such, repurposing drugs are being used as viable therapeutic options due to the limited effective treatments for Long COVID symptoms. Ivermectin is one of the emerging repurposed drugs that has been shown effective to have antiviral effects in clinical trials. In addition, antioxidant compounds are also gaining attention due to their capabilities of reducing inflammation and severity of symptoms. Due to the absence of knowledge in pharmacogenomics and modes of actions in the human body for these compounds, this study aims to provide a pharmacogenomic profile for the combination of ivermectin and six selected antioxidants (epigallocatechin gallate (EGCG), curcumin, sesamin, anthocyanins, quercetin, and N-acetylcysteine (NAC)) as potentially effective regimens for long COVID symptoms. Results showed that there were 12 interacting genes found among the ivermectin, 6 antioxidants, and COVID-19. For network pharmacology, the 12 common interacting genes/proteins had the highest associations with Pertussis pathway, AGE-RAGE signaling pathway in diabetic complications, and colorectal cancer in the Kyoto Encyclopedia of Genes and Genomes (KEGG) analyses. Disease analyses also revealed that the top three relevant diseases with COVID-19 infections were diabetes mellitus, ischemia, reperfusion injury. We also identified 6 potential target microRNAs (miRNAs) of the 12 commonly curated genes used as molecular biomarkers for COVID-19 treatments. The established pharmacogenomic network, disease analyses, and identified miRNAs could facilitate developments of effective regimens for chronic sequelae of COVID-19 especially in this post-pandemic era. However, further studies and clinical trials are needed to substantiate the effectiveness and dosages for COVID-19 treatments.

## 1. Introduction

Exploration for preventive medications or effective regimens for sequelae post infections of severe acute respiratory syndrome coronavirus 2 (SARS-CoV-2) with well-evidenced safety or efficacy has been a critical issue since the pandemic onset. Pharmacogenetic data for coronavirus disease 2019 (COVID-19) drugs could be essential in establishing personal profiles of bodily responses to medications and could be adopted as a strategy to prevent potential adverse drug reactions in different individuals [1]. Ivermectin, originally serving as an antiparasitic agent, has emerged as a repurposed medicine for long COVID resolutions because of the well-evidenced antiviral activities on various RNA viruses [2]. Numerous studies including clinical trials and meta-analyses have been showing the effectiveness of ivermectin on the symptom resolution of SARS-CoV-2 [3,4]. However, except for the increasing evidence from pre-clinical or clinical studies, there have been no thorough pharmacogenetic assessment to explore drug–gene, drug–protein, and drug–disease interactions to dissect antiviral mechanisms of ivermectin in human body. Pharmacogenetic analyses of ivermectin could provide clear modes of action for the repurposing of this drug in alleviating COVID-19 symptoms and more proper administration suggestions could be constructed.

On the other hand, several studies have connected the associations between the progression or severity of COVID-19 symptoms and the overproduction of reactive oxygen species (ROS) and disruptions of antioxidant systems [5,6]. One of the major complications of SARS-COV-2 is the acute respiratory distress syndrome (ARDS), which could be caused by the excessive production of pro-inflammatory cytokines (cytokine storm syndromes) such as interleukin (IL)-6 [5,6]. High levels of cytokines indicate poor prognosis in COVID-19, and increasing studies have demonstrated that a cytokine storm could contribute to mortality in COVID-19 patients. Also, interconnected pathways have suggested the relationship between oxidative damage and inflammation resulted from COVID-19 infections, leading to the application of antioxidants in attenuating oxidative stress or inflammation effects as a potentially preventive measure and medication for long COVID [5].

Therefore, this study aims to provide deep pharmacogenetic analyses of ivermectin and explore the common mechanisms of this repurposing drug and six selected antioxidants including the epigallocatechin gallate (EGCG), curcumin, sesamin, anthocyanins, quercetin, and N-Acetylcysteine (NAC) in attenuating COVID-19 symptoms. The six antioxidants were selected based on the scientific evidence of their antioxidant effects which are associated with antiviral properties, including the inhibition of SARS-CoV-2 replication and the alleviation of its symptoms [7,8,9,10,11,12].

In addition to the increasing clinical evidence, understanding the antiviral mechanisms of ivermectin and the anti-inflammatory effects of the investigated antioxidants before an overt cytokine storm could be crucial in designing effective administrative combinations of beneficial compounds to tackle Long COVID symptoms. Taken together, the objectives of this study are threefold: (i) screening the interacting genes among ivermectin, six selected antioxidants, and COVID-19, (ii) exploring the drug–gene, drug–proteins, and drug–disease associations with thorough pharmacogenomic analyses of the interacting genes, and (iii) establishing pharmacogenomic networks of the investigated compounds to imply the potential modes of action of their involvements in alleviating oxidative stress or antiviral effects in response to the long COVID.

## 2. Results

### 2.1. Structural Formulas of Ivermectin and the 6 Selected Antioxidants

**Figure 1:**
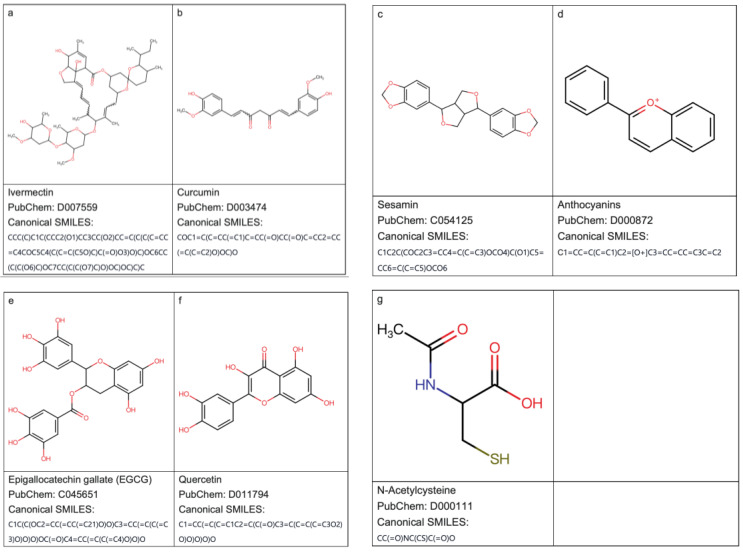
The chemical structures of (**a**) ivermectin and the six investigated antioxidants including (**b**) curcumin, (**c**) sesamin, (**d**) anthocyanins, (**e**) epigallocatechin gallate (EGCG), (**f**) quercetin, and (**g**) N-acetylcysteine (NAC). Chemical structures were visualized using SwissBioisostere (http://www.swissbioisostere.ch/ (accessed on 27 August 2023)) [13] from Canonical SMILES.

The chemical structures of ivermectin and the six investigated antioxidants including epigallocatechin gallate (EGCG), curcumin, sesamin, anthocyanins, quercetin, and N-acetylcysteine (NAC) are presented (Figure 1).

### 2.2. Interacting Genes of Ivermectin and the Six Selected Antioxidants with COVID-19

**Figure 2:**
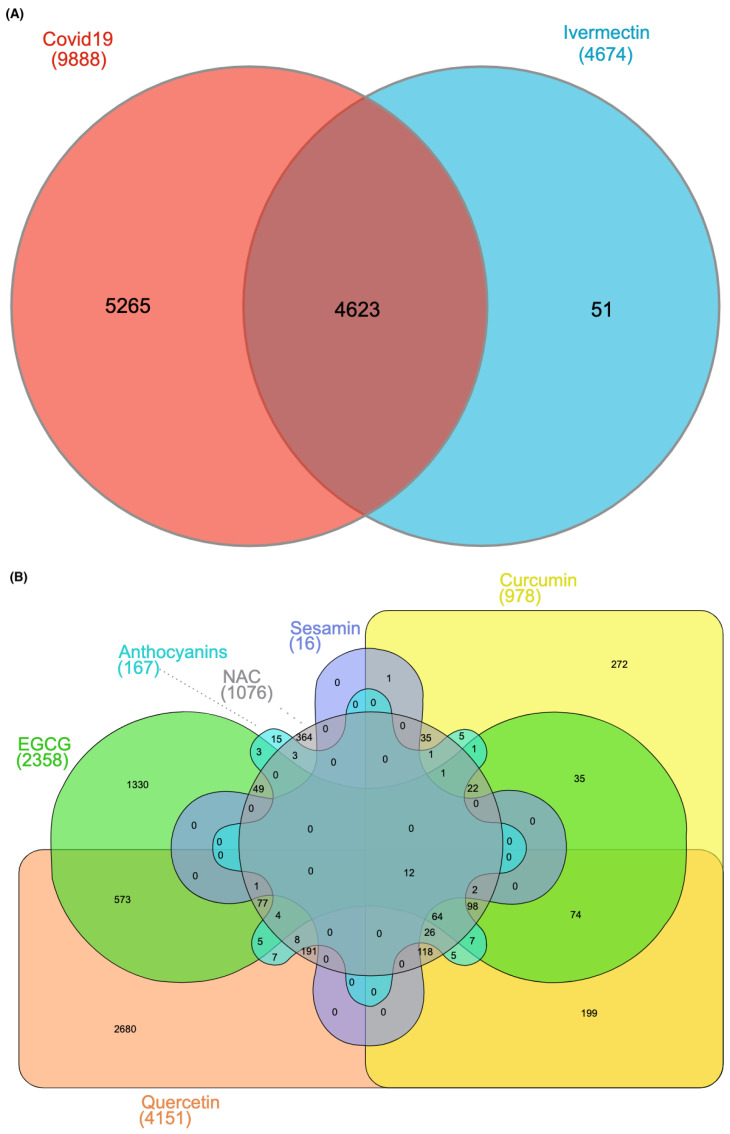
Venn diagram of the interacting genes between (**A**) ivermectin and COVID-19, or (**B**) the six investigated antioxidants and COVID-19.

To understand the relationship between genes and drug responses, we explored chemical–gene and COVID-19–gene interactions from the CTD database. Curated interacting genes of ivermectin or the six investigated antioxidants and COVID-19 were explored using InteractiVenn [14] and visualized with Venn diagrams (Figure 2). Results showed that there are 4623 interacting genes between the ivermectin and COVID-19 (Figure 2A; Supplementary Table S1). For the 6 investigated antioxidants, there are 2358, 978, 16, 167, 4151, and 1076 interacting genes for the epigallocatechin gallate (EGCG), curcumin, sesamin, anthocyanins, quercetin, and N-Acetylcysteine (NAC), respectively (Figure 2B; Supplementary Table S1). Finally, 12 interacting genes were found between ivermectin, the 6 antioxidants, and COVID-19 (Figure 2B; Table 1). 

### 2.3. Gene Ontology Analyses

**Figure 3:**
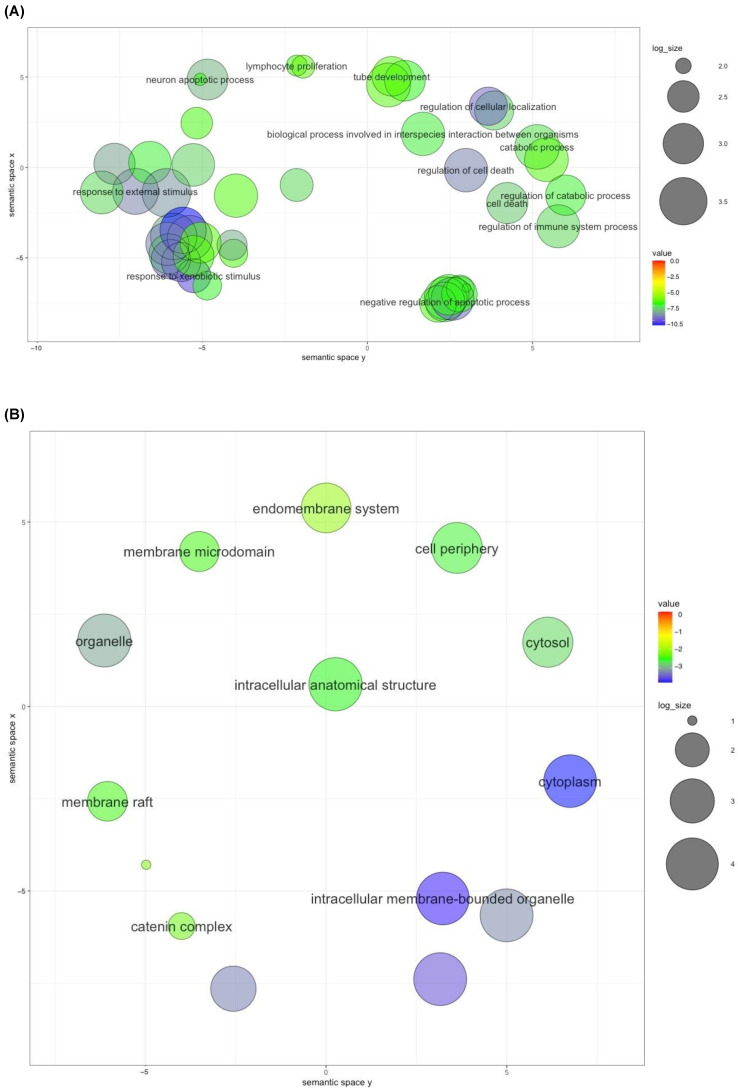

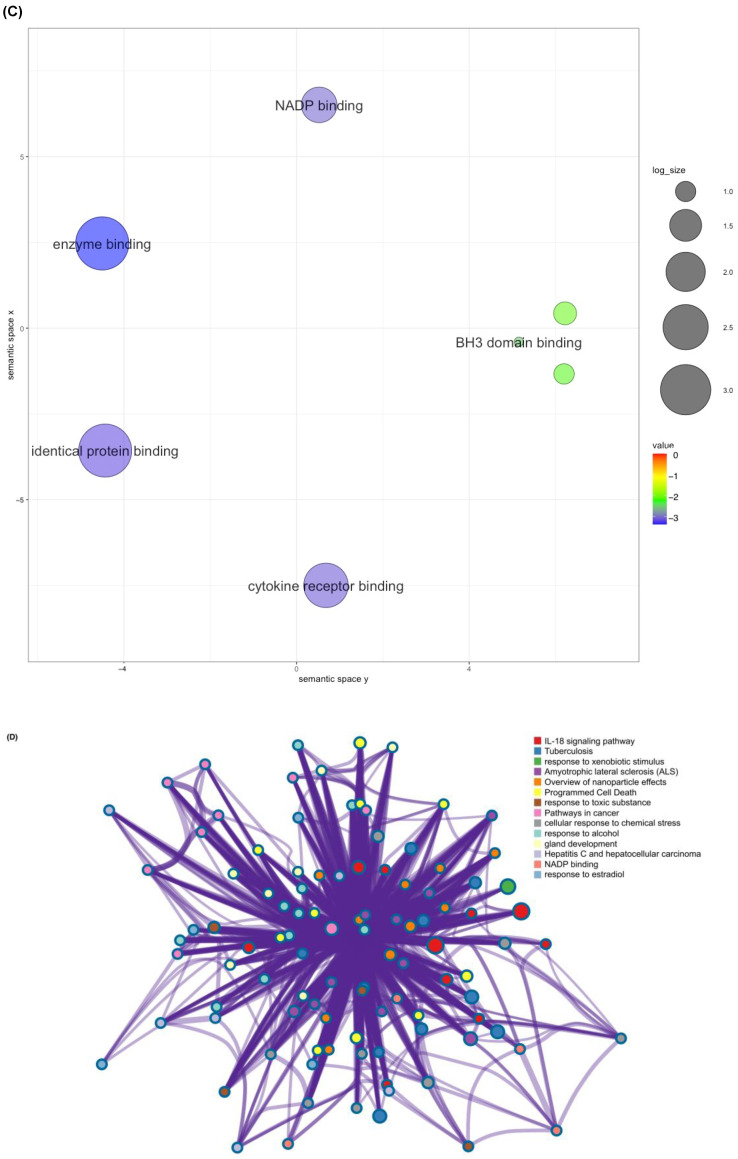
Gene ontology analyses of (**A**) biological process (BP), where circle size and color indicate the number of occurrences of GO terms in the secreted gene set annotation list, (**B**) cellular component (CC), and (**C**) molecular function (MF) scatterplot. (**D**) Ontology enrichment clustering network. Cluster annotations are shown in color code.

To identify the biological processes, cellular locations, and molecular functions of the 12 genes targeted by ivermectin and the 6 selected antioxidants interacting with COVID-19, and gene ontology analyses were conducted. Enriched terms were summarized by the GO terms’ semantic similarities in a two-dimensional scatterplot for the three gene ontology domains (CC, cellular component, MF, molecular function, and BP, biological process). For biological process, the final terms presented were screened and selected based on the adjusted *p*-values (Figure 3A). The scatterplot contains responses from the 12 interacting genes to the xenobiotic stimulus (GO:0009410), regulation of cellular localization (GO:0060341), regulation of cell death (GO:0010941), response to external stimulus (GO:0009605), cell death (GO:0008219), regulation of immune system process (GO:0002682), catabolic process (GO:0009056), negative regulation of apoptotic process (GO:0043066), biological process involved in interspecies interaction between organisms (GO:0044419), regulation of catabolic process (GO:0009894), neuron apoptotic process (GO:0051402), lymphocyte proliferation (GO:0046651), and tube development (GO:0035295) related Gene Ontologies (Figure 3A; Supplementary Table S2).

For the cellular component, it was revealed that cytoplasm (GO:0005737), intracellular membrane-bounded organelle (GO:0043231), organelle (GO:0043226), cytosol (GO:0005829), cell periphery (GO:0071944), intracellular anatomical structure (GO:0005622), membrane raft (GO:0045121), membrane microdomain (GO:0098857), catenin complex (GO:0016342), and endomembrane system (GO:0012505) related Gene Ontologies (Figure 3B; Supplementary Table S2). For molecular function, the GO terms scatterplot reveals a stronger occurrence of GO terms corresponding to enzyme binding (GO:0019899), identical protein binding (GO:0042802), cytokine receptor binding (GO:0005126), NADP binding (GO:0050661), and BH3 domain binding (GO:0051434) GO related (Figure 3C; Supplementary Table S2). In addition, the enrichment network visualization was shown with the intra- and inter-cluster similarities of the enriched terms based on the subset of representative terms from the full cluster (Figure 3D). 

### 2.4. Target Gene–KEGG Pathway Network

**Figure 4:**
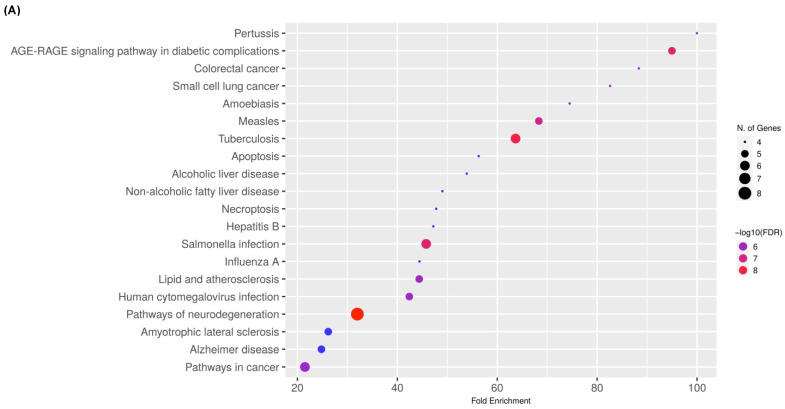

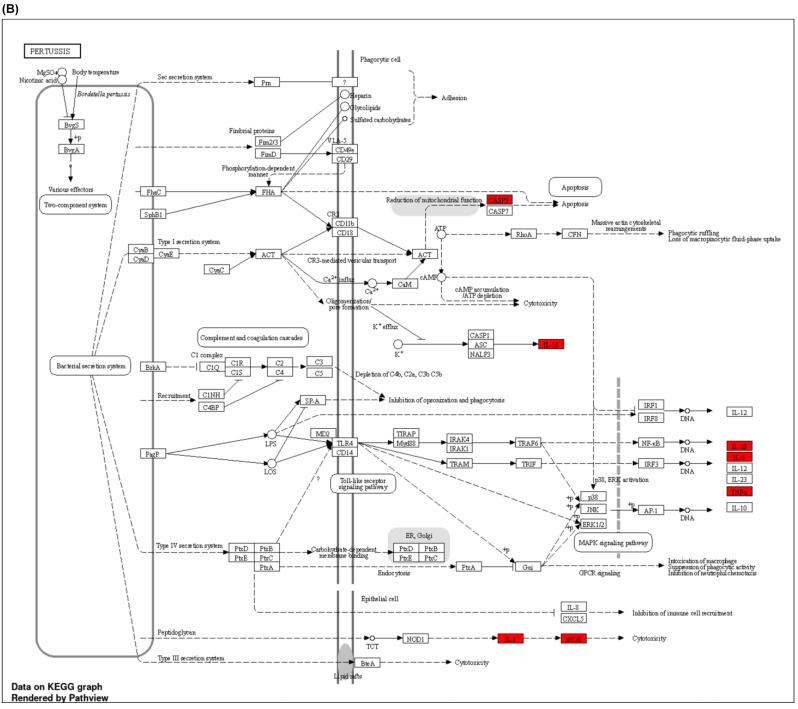

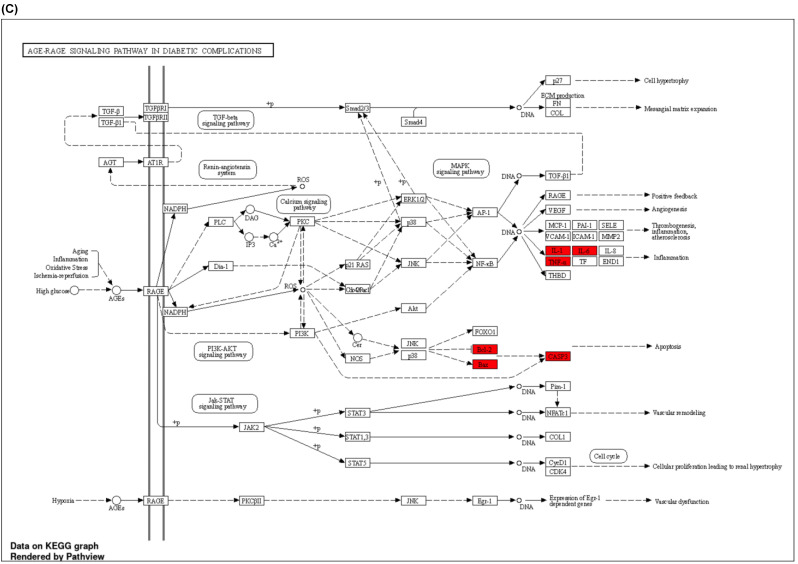
(**A**) KEGG pathway enrichment analysis for the target 12 genes. The gene ratios refer to the ratio of enriched genes to all target genes, and counts refer to the number of enriched genes. KEGG pathway analyses for the (**B**) Pertussis pathway (hsa05133) and (**C**) AGE-RAGE signaling pathway in diabetic complications (hsa04933).

To construct the target gene–KEGG pathway networking, the network was formed by the 12 hit genes targeted by the ivermectin and the 6 selected antioxidants interacting with COVID-19 by using the KEGG database. The top three KEGG pathways was the Pertussis pathway (hsa05133), AGE-RAGE signaling pathway in diabetic complications (hsa04933), and colorectal cancer (hsa05210) (Figure 4A; Supplementary Table S3). The pathway analyses were established with the KEGG diagram by focusing on the top three signaling pathways. For the Pertussis pathway, the main responsible genes for the pathways were CASP3, IL-1β, IL-1, IL-6, TNFα, and iNOS (Figure 4B). For the AGE-RAGE signaling pathway in diabetic complications, the main responsible genes for the pathways were IL-1, IL-6, TNFα, Bcl-2, Bax, and CASP3 (Figure 4C). 

### 2.5. Network Analysis

**Figure 5:**
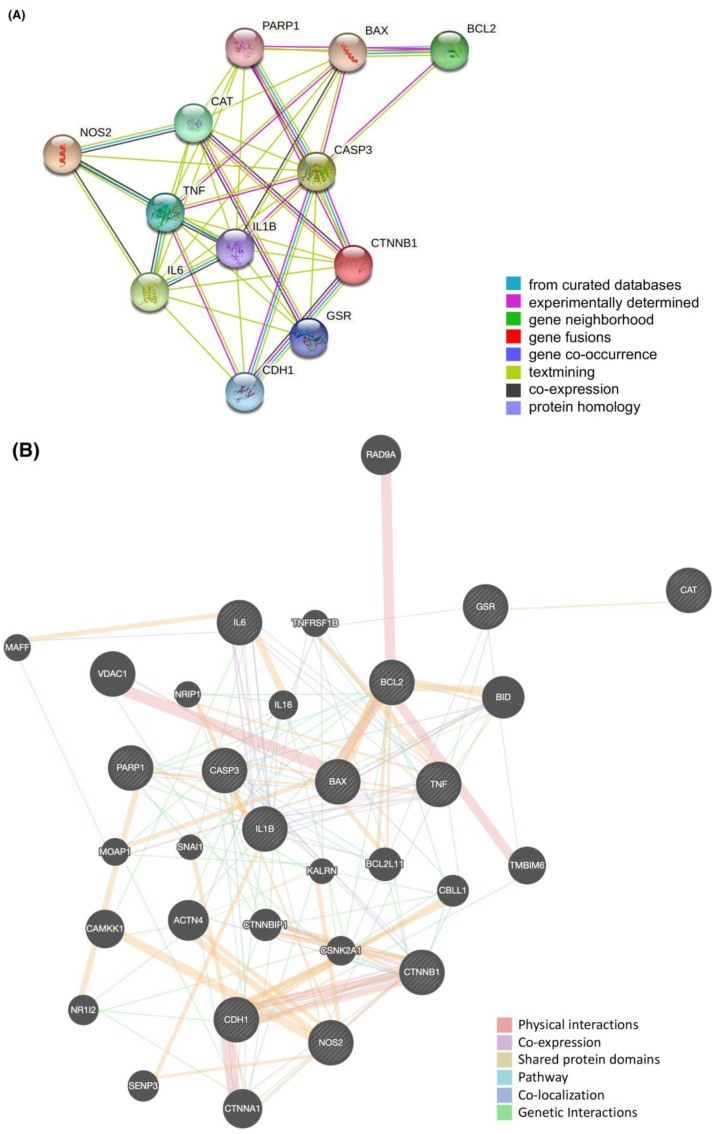
The PPI networks of the 12 common curated genes/proteins generated from the databases of (**A**) STRING and (**B**) GeneMANIA.

The interactions among the 12 common genes/proteins were generated by applying the online complex protein database, STRING. The PPI network was adjusted with the medium confidence interaction score of 0.4 and built with 12 nodes and 45 edges with an average node degree of 7.5 and an average clustering coefficient of 0.836. The PPI enrichment was considered significant at *p*-value = 5.55 × 10^−9^ < 0.01 (Figure 5A). In addition, we used another database to explore protein–protein interactions among the 12 common genes/proteins in the GeneMANIA website by automatically selecting weighting method with 20 related genes, 32 total genes, and 179 total links. Results showed that the interconnections between proteins were physical interaction, co-expression, shared protein domains, pathway, co-localization, and genetic interaction (Figure 5B). 

### 2.6. Disease Analysis

**Figure 6:**
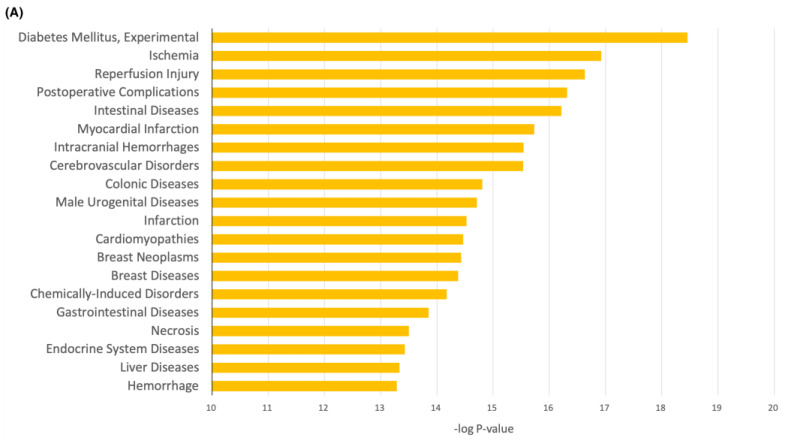

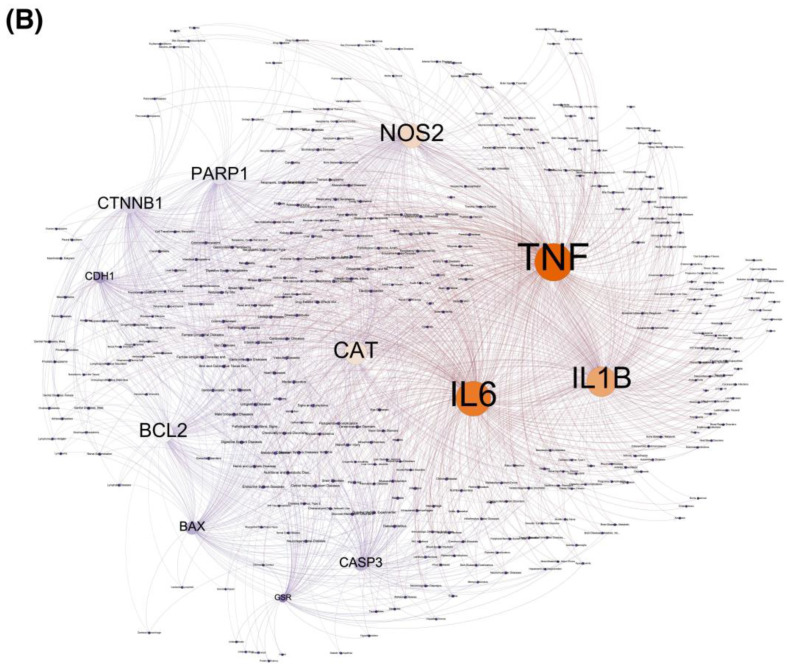
(**A**) Disease analysis and (**B**) the gene–disease network of ivermectin and the 6 investigated antioxidants and COVID-19.

The 12 common genes/proteins from ivermectin and the 6 investigated antioxidants were fully analyzed for associated human diseases inferred by the CTD perform analyses (Figure 6A). Results showed that the top ten diseases respond to COVID-19 and ivermectin and the six investigated antioxidants were diabetes mellitus (experimental), ischemia, reperfusion injury, postoperative complications, intestinal diseases, myocardial infarction, intracranial hemorrhages, cerebrovascular disorders, colonic diseases, and male urogenital diseases (Figure 6A; Supplementary Table S4). The functional enrichment analyses for the disease–gene associations of the 12 most frequently curated ivermectin, the 6 investigated antioxidants and the COVID-19 related genes were also analyzed (Figure 6B; Supplementary Table S4). Results showed that the most curated genes were TNF, IL6, and IL1B.

### 2.7. Target Identification of Genes

**Figure 7:**
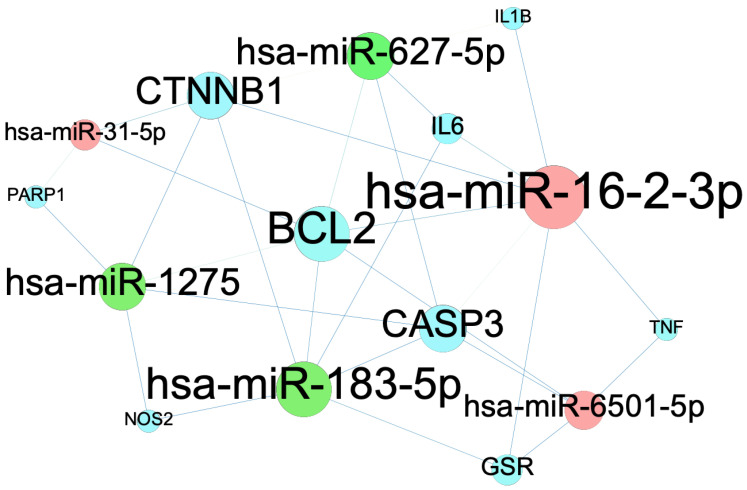
Potential targets of miRNAs to the 12 common curated genes.

The target genes of the miRNAs are as follows: the 7 target genes (*TNF*, *CASP3*, *IL1B*, *BCL2*, *IL6*, *GSR*, and *CTNNB1*) of the hsa-miR-16-2-3p microRNA, the 6 target genes (CASP3, NOS2, BCL2, IL6, GSR, and CTNNB1) of the hsa-miR-183-5p, the 4 target genes (TNF, CASP3, BCL2, and GSR) of the hsa-miR-6501-5p microRNA, the 5 target genes (CASP3, IL1B, BCL2, IL6, and CTNNB1) of the hsa-miR-627-5p microRNA, the 3 target genes (BCL2, PARP1, and CTNNB1) of the hsa-miR-31-5p microRNA, and the 5 target genes (CASP3, NOS2, BCL2, PARP1, and CTNNB1) of hsa-miR-1275 microRNA were found from the 12 commonly curated genes set individually. The network was visualized using Gephi, with blue representing target genes, red representing up-regulated microRNAs, and green representing down-regulated microRNAs (Figure 7; Table 2).

## 3. Discussion

### 3.1. Ivermectin as a Repurposing COVID-19 Drug

Ivermectin is an FDA-approved anti-parasitic drug which was previously evidenced to have broad-spectrum anti-viral activity in vitro. It possesses many potential effects to treat various diseases with its antimicrobial, antiviral, and anti-cancer properties. It has been proven that ivermectin has high antiviral effects on RNA viruses such as Zika, dengue, yellow fever, West Nile, Hendra, Newcastle, Venezuelan equine encephalitis, chikungunya, Semliki Forest, Sindbis, Avian influenza A, Porcine Reproductive and Respiratory Syndrome, Human immunodeficiency virus type 1, and severe acute respiratory syndrome coronavirus. In addition to RNA viruses, several studies also found its antiviral effects on DNA viruses including Equine herpes type 1, BK polyomavirus, pseudorabies, porcine circovirus 2, and bovine herpesvirus [2].

Novel experimental evidence has also demonstrated that ivermectin is active and efficacious in vitro against Chagas disease, Leishmaniases, arboviruses, and SARS-CoV-2 [15]. Hariyanto et al. [3] also evidenced through a meta-analyses that ivermectin administration had an association with favorable outcomes of COVID-19, compromising of a higher rate of negative RT-PCR test results, shorter time to achieve negative RT-PCR test results, higher rate of symptom alleviation, shorter time to symptom alleviation, shorter time to hospital discharge, and a reduction in the severity and mortality rate from COVID-19. Moreover, the mixture of homologues called ivermectin (avermectin-B1a + avermectin-B1b) has been shown to be a Multi-Target Drug (MTD) with potential antiviral activity against SARS-CoV-2 in vitro. The homologues were found to be able to slightly modify the conformation and stability of the connection points between the Cα atoms of the residues that make up the structural network of proteins (nodes), compared to free proteins. Each homologue was able to differently modify the distribution of the quasi-rigid regions of the proteins, which could theoretically alter their biological activities. These results could provide a biophysical–computational view of the potential MTD mechanism that has been reported for ivermectin [16].

The effective antiviral effects of ivermectin have made it a potential candidate in the treatment of SARS-CoV-2 as well as other types of positive-sense single-strand RNA viruses. Although in vivo studies revealed a broad range of the antiviral effects of ivermectin, clinical trials are of great essentiality to appraise the efficacy of ivermectin in the human body [2]. More randomized clinical trial studies are suggested to be done for confirmation of safety and efficacy of the ivermectin. Also, ivermectin could be considered an essential drug for future COVID-19 therapy models [17]. Biswas et al. [1] also strongly suggested undertaking a pharmacogenetic assessment for the drug–gene pairs (ivermectin-ABCB1) in COVID-19 patients for advancing precision medicine. Molecular docking and computational studies are also promising tools to achieve new therapeutics against SARS-CoV-2 infection.

In addition, Ahmed et al. [18] identified 17 drug target proteins (SMAD4, GSK3B, SIRT1, ATM, RIPK1, PRKACB, MED17, CCT2, BIRC3, ETS1, TXN, FOXC1, GATA2, YY1, FOXL1, TP53, and SRF) from a large number of alternatives based on microarray gene-expression profiles of SARS-CoV-1 infected and control samples. The proposed target proteins guided the top-ranked 7 repurposing drugs (Rapamycin, Tacrolimus, Torin-2, Redotinib, Ivermectin, Danoprevir, and Daclatasvir) were identified for the treatment against SARS-CoV-1infections. As a matter of fact, it would be of great importance to explore more of the pharmacogenomic mechanism of ivermectin as one of the crucial emerging repurposing drugs.

### 3.2. Antioxidants in Attenuating COVID-19 Symptoms

The drug discovery and clinical development for COVID-19 have been mainly focused on monotherapies. Combination therapies have the advantages of increasing potency of individual compounds and combat the rapid evolution of resistance. It would be of interest to explore the combination effects of ivermectin along with the antioxidants with well-evidenced antiviral effects [19]. There have been many antioxidant compounds adopted for attenuations of long COVID symptoms. This study selected those that have been frequently discussed and applied since COVID-19 pandemic, including the Epigallocatechin Gallate (EGCG), curcumin, sesamin, anthocyanins, quercetin, and N-Acetylcysteine (NAC).

Epigallocatechin Gallate (EGCG) is a polyphenolic compound found in green tea that has recently been investigated as a potential antiviral agent. Studies have shown that EGCG has antiviral properties that could be used to inhibit infection of the novel SARS-CoV-2 virus [7]. Studies on EGCG have demonstrated that it can inhibit the replication and assembly of the SARS-CoV-2 virus. In particular, EGCG has been shown to inhibit the viral replication cycle by blocking the interaction between the viral RNA and the viral replication machinery [20,21]. This could help reduce the spread of the virus and potentially lead to new treatment strategies. In addition, EGCG has been shown to have anti-inflammatory properties and can help reduce the severity of symptoms associated with the virus [22]. This could be useful for those already infected with the virus, as it could reduce the severity of their symptoms and help them recover faster [22].

On the other hand, the potential of curcumin to inhibit infection of live SARS-CoV-2 has been the subject of much research in recent years. Curcumin, a natural compound derived from the spice turmeric, has been studied for its anti-inflammatory, antioxidant, and antiviral properties [8]. It has been found to possess the ability to inhibit the replication of a wide range of viruses, including SARS-CoV-2. Studies have suggested that curcumin can act on the different steps of the virus replication cycle, including inhibition of viral entry into the cell, inhibition of viral protein synthesis, and inhibition of viral assembly and release [8,23]. In addition, it is believed to possess immunomodulatory properties that can help to regulate the immune system and reduce inflammation [24]. In terms of its efficacy against SARS-CoV-2, research has shown that curcumin can significantly reduce the infectivity of the virus in cultured cells. It has also been shown to decrease the production of the virus in infected cells [25]. Furthermore, studies have demonstrated that curcumin-piperine co-supplementation in outpatients with COVID-19 could significantly reduce weakness in a randomized double-blind, placebo-controlled trial [26].

The potentiality of sesamin as a compound to inhibit the infection of live SARS-CoV-2 is an exciting development in the fight against the virus [9]. Sesamin, as a major compound in the *Paulownia tomentosa*, is a naturally occurring compound found in sesame seeds and has been recently reported to possess potent antiviral activity against SARS-CoV-2 in vitro [27,28]. Studies have shown that sesamin is able to bind to the spike protein of the virus, preventing it from entering the host cell. This creates a blockade which effectively stops the virus from replicating and ultimately reduces the chance of infection [24]. The antiviral activity of sesamin against SARS-CoV-2 has been tested in animal models and the results showed promising effects. The compound was able to reduce the viral load and decrease the severity of symptoms in infected animals [27]. This suggests that sesamin could be used as a treatment for SARS-CoV-2, either as a supplement or as part of a combination therapy.

Anthocyanins are a type of flavonoid compound that has been studied for its potential to inhibit the infection of live SARS-CoV-2 [10].

Anthocyanins have been found to have antiviral, anti-inflammatory, and antioxidant properties, which may be beneficial in preventing the spread of the virus. Studies have shown that anthocyanins can bind to the virus’ surface proteins and disrupt its ability to enter and infect host cells [29]. Additionally, it was found that anthocyanins could inhibit the nuclear factor-κB (NF-κB) pathway by regulating the miR-138-5p/sirtuin-1 (SIRT1) axis, thus inhibiting airway inflammation in asthmatic mice [30]. While there is promising research into the potential of anthocyanins in inhibiting the infection of live SARS-CoV-2, more studies are needed to determine the exact extent of their effectiveness and the most suitable dose for humans. In addition, more research is needed to determine if anthocyanins can be used in conjunction with other antiviral agents to enhance their effectiveness. Overall, anthocyanins may offer a promising potential for inhibiting the infection of live SARS-CoV-2. More research is needed to determine the safety and effectiveness of this compound and to understand the best way to use it to help prevent the spread of this virus.

Quercetin is a plant-based flavonoid that has been studied for its potential to inhibit infection of live SARS-CoV-2. Manjunath and Thimmulappa [11] found that quercetin may inhibit SARS-CoV-2 entry into cells by altering viral envelope proteins and inhibit SARS-CoV-2 replication by activating the NRF2 pathway. Attenuations of proinflammatory signals and cytokine release syndrome and reduction of coagulopathy by inhibiting protein disulphide isomerase were also observed. While quercetin has been studied in vitro, there is limited evidence of its effectiveness in vivo. It was found that quercetin as a compound with anti-inflammatory, antioxidant, and analgesic functions and could act as a NLRP3 inflammasome inhibitor can be a potential treatment for severe inflammation in patients with life-threatening conditions from COVID-19 infection [31]. Di Pierro et al. [32] also found quercetin could statistically shorten the timing of molecular test conversion from positive to negative and reduce symptoms severity and negative predictors of COVID-19 in a 2-week, randomized, open-label, and controlled clinical study. Quercetin is available over-the-counter in many forms, including capsules and powders. While it is generally considered to be safe, the public should always consult with their doctor before taking any supplements. It is also important to note that taking too much quercetin can cause side effects, so it is important to follow the instructions on the package carefully. Overall, it is clear that quercetin shows potential as an inhibitor of SARS-CoV-2 [11,31,32].

N-Acetylcysteine (NAC) is a compound that has recently been studied for its potential to inhibit infection of live SARS-CoV-2. It was found that a shorter length of hospital stay (LOS) was observed in patients receiving NAC with SARS-CoV-2 pneumonia when compared to those who did not receive NAC [12]. NAC is a naturally occurring compound that is able to boost the body’s ability to fight off infection and has been found to be effective in reducing symptoms in some patients with respiratory illnesses [33]. Poe and Corn [34] found that NAC could reduce the amount of SARS-CoV-2 that could attach to the ACE2 receptors on human cells, thus reducing the virus’ ability to infect the cells. Another study found that NAC was able to reduce the replication and spread of SARS-CoV-2 in cell cultures [35]. These results suggest that NAC may be an effective inhibitor of SARS-CoV-2 infection. However, more research is needed to fully understand the potential of NAC as an inhibitor of SARS-CoV-2 infection. Clinical trials are needed to determine the safety and efficacy of NAC in humans, as well as to understand the optimal dosage and duration of treatment [36]. It is also important to investigate potential side effects of NAC, as some studies have suggested that it may cause liver damage or other adverse reactions [37]. Overall, NAC appears to be a promising compound for inhibiting infection of live SARS-CoV-2. However, more research is necessary to fully understand the potential of NAC as an inhibitor of SARS-CoV-2 infection and to determine the safety and efficacy of NAC in humans [33].

Taken together, due to the fact that oxidation, inflammation, and immune response impairment are interconnected and play as key determinants in COVID-19, investigations of the modes of action of antioxidant effects of these compounds in the pharmacogenetic perspective would be of great interest for designing personalized medicines [5]. Moreover, higher levels of antioxidants in COVID-19 patients were found to be beneficial to prevent disease progression in a systematic research evaluating association between antioxidants and COVID-19 outcomes [38]. However, further research is needed to fully understand the effectiveness and appropriate dosages of antioxidants in the context of COVID-19 treatment.

### 3.3. Pharmacogenomic Analyses of Ivermectin and the Selected 6 Antioxidants

Pharmacogenomics is an important area of research that can significantly improve the efficacy of drug therapies [39]. Network analyses of pharmacogenomics of ivermectin and the selected antioxidants can be used to identify the genetic determinants of drug response [40]. Such analyses can provide insights into the molecular mechanisms underlying drug/compound responses and can help to identify individuals who are more likely to respond favorably to the drug.

To explore pharmacogenomics of ivermectin and the 6 selected antioxidants, gene expression profiling, gene network analysis, and gene-set enrichment analysis were obtained to identify gene–gene or protein–protein interactions among the 12 common genes/proteins targeted interacting with COVID-19. Gene network analysis can be used to identify pathways or networks that are associated with drug/compound response, while gene-set enrichment analysis can be used to identify sets of genes that are associated with responses of ivermectin and the selected antioxidants. For biological processes in gene ontology analyses, consistent with previous studies, processes including the regulation of cell death (GO:0010941) and regulation of immune system process (GO:0002682) were associated with COVID-19 [41,42]. For the KEGG pathway network, Pertussis pathway (hsa05133), AGE-RAGE signaling pathway in diabetic complications (hsa04933), and colorectal cancer (hsa05210) were also evidenced to be correlated with COVID-19 in several clinical studies [43,44,45]. For the protein–protein interactions, we identified that physical interaction, co-expression, shared protein domains, pathway, co-localization, and genetic interaction were all included among the12 common proteins targeted by the ivermectin and the 6 selected antioxidants interacting with COVID-19. The results are comparable with another research studying another COVID-19 drug (Bevacizumab) that there were 61.00% of co-expression, 16.37% physical interaction, 10.78% of the pathway, 8.87% of prediction, 6.09% genetic interactions, 3.07% co-localization, and 3.88% of shared protein domains based on the analyses of protein–protein interactions [46].

For disease analyses, it was found that the 12 common genes/proteins from ivermectin and the 6 investigated antioxidants are associated with the top three diseases including diabetes mellitus (experimental), ischemia, and reperfusion injury. In accordance with the results of disease analysis in this study, Lim et al. [47] found there are potential pathogenetic links between COVID-19 and diabetes mellitus include effects on glucose homeostasis, inflammation, altered immune status and activation of the renin-angiotensin-aldosterone system (RAAS). It was also suggested that SARS-CoV-2 infection is associated with a high risk for thrombotic complications, including the acute limb ischemia (ALI) [48]. Ashraf et al. [49] linked ischemia-reperfusion (IR) injury to multiple organ damage by SARS-CoV-2, suggesting COVID-19 may cause a reduction in oxygen towards an organ, which leads to IR injury.

### 3.4. microRNA Analyses for COVID-19 Medicines

We identified 6 microRNAs (miRNAs) based on the connections with the 12 common genes/proteins from ivermectin and the 6 investigated antioxidants [50,51]. The miRNAs are single-stranded noncoding RNAs which are capable of inhibiting gene translations and downregulating target gene expressions by binding to the 3′-untranslated regions (3′-UTRs) of mRNAs [52]. The miRNAs could play essential roles in a variety of biological processes including development, differentiation, regulation, and stress of inflammation in the host immune cells. It was also found that miRNAs are master regulators of the pathways involved in asthma [53].

Increasing evidence has demonstrated that microRNA serves as a crucial role in suppression of many COVID-19 related genes, among which the miR-223 plays as a key role in the process of COVID-19 immunopathogenesis. The miR-223 is a hematopoietic cell-derived miRNA which was revealed to be associated with regulating monocyte-macrophage differentiation, neutrophil recruitment, and pro-inflammatory responses. It controls inflammation by targeting a variety of factors, including TRAF6, IKKα, HSP-70, FOXO1, TLR4, PI3K/AKT, PARP-1, HDAC2, ITGB3, CXCL2, CCL3, IL-6, IFN-I, STMN1, IL-1β, IL-18, Caspase-1, NF-κB, and NLRP3. Numerous studies also reported the role of miR-223 in inhibiting inflammation to prevent tissue damage during infection and other inflammatory diseases. Important targets for miR-223 involved in infection and inflammation are NLRP3, IKKα, and NF-κB. The NF-κB and NLRP3 inflammasome. The miR-223 was also found to be capable of targeting viruses directly [54].

### 3.5. Essentiality and Limitation of Pharmacogenomic for COVID-19 Treatments

Although the virological features and genomic sequence of SARS-CoV-2 have been identified, the mechanisms associated with immunopathogenesis of COVID-19 still remain unclear. Increased application of repurposed drugs without established efficacy has been a concerning issue during or post COVID-19 pandemics for treating various long COVID symptoms. Advancements in drug development also seem to barely keep up with the mutation rates of SARS-CoV-2. Drug repurposing could be an essential and attractive approach during emergent clinical applications with its advantages in less time or budget needed [55]. However, pharmacogenetic approaches including drug–gene or drug–drug interactions, drug toxicity, and patient comorbidities should be re-considered for more effective treatments of repurposing drug in clinical studies/trials [1,55]. Biswas et al. [1] also strongly suggested pharmacogenetic assessments through molecular docking or computational approaches for drug–gene pairs of ivermectin-ABCB1 to achieve precision medicines in COVID-19 patients.

One of the implementation challenges of pharmacogenomics is to investigate how the genes influence individuals’ responses to pharmacological treatments (e.g., minimal dosage for efficacy, and adverse effects) since usually more than one gene is involved in the drug response pathway [38]. Recently, data of 132 pharmacogenomic dosing guidelines for 99 drugs are available from organizations such as the Clinical Pharmacogenetics Implementation Consortium (CPIC) and the Dutch Pharmacogenetics Working Group (DPWG) [38]. In clinical studies, the role of pharmacogenetic analysis is to design personalized medication to optimize drug therapy by ensuring maximum efficacy and minimal adverse effects based on a patient’s genotype [56,57]. Also, there are regulatory bases for the implementation of good pharmacogenomic practice [58]. Therefore, minimal dosage suggestions for specific drug in medication could be various among patients. The network/disease analysis based on the interplay of the 12 genes is also contributed from the ivermectin and 6 antioxidants. Further information of differentiating efficacy of specific compound on particular disease and the starting doses should be provided by pharmacokinetic/pharmacodynamic analysis.

## 4. Materials and Methods

### 4.1. Curated Interaction Analysis

The Comparative Toxicogenomics Database (CTD; Department of Bioinformatics, The Mount Desert Island Biological Laboratory, Salisbury Cove, ME, USA; http://ctd.mdibl.org (accessed on 12 June 2022)) was employed to analyze curated interactions of ivermectin and the six selected antioxidants, epigallocatechin gallate (EGCG), curcumin, sesamin, anthocyanins, quercetin, and N-Acetylcysteine (NAC), and COVID-19. The CTD is a powerful database that is capable of exploring interactions between chemical exposures and biological responses [59]. It can also provide information including chemical–gene/chemical–protein interaction, and chemical–disease/gene-=–disease relationships.

### 4.2. Gene Ontology (GO) Enrichment Analysis

For GO mapping, the GO terms for the 12 target genes based on homologies were extracted (http://www.geneontology.org (accessed on 27 August 2023)). The semantic similarities among the enriched Gene ontology (GO) terms (*p* value  <  1%) using Gene Set Analyzer were analyzed by CTD (http://ctdbase.org/) and visualized by REVIGO (version 1.8.1; http://revigo.irb.hr/), incorporated with the R software (version 3.3.3; https://www.r-project.org/ (accessed on 27 August 2023)). Gene Ontology scatterplots were constructed by using REVIGO [60] in R for all GO terms and genes within the 12 identified target genes. Settings used for REVIGO program were as follows, database: Homo sapiens, semantic similarity: 0.7 (medium), and semantic similarity measure: SimRel.

The scatter plots represent the summarized GO term analysis of the 12 target gene set genes. Semantically similar GO terms were plotted near to each other on the (unit-less) *X*-*Y* axes, such that functionally similar terms were located nearby, and more unrelated terms were further apart in space. The size of the individual bubbles indicates the frequency of the GO term in the database, in which larger bubbles reveal more general terms and smaller bubbles are more specific terms. The color indicates the *p*-value of the enrichment of each GO term.

Furthermore, we selected a subset of representative terms from the full cluster and converted them into a network layout. We used KEGG (http://www.kegg.jp/ or http://www.genome.jp/kegg/), an encyclopedia of functional genes and genomes, to explore the networks of molecular interactions, reactions and relations in the form/map of KEGG pathways [61].

Visualization was conducted using ShinyGO version 0.77 [62] and Metascape [63], and further rendered in Cytoscape version 3.9.1 [64] using a "force-directed" layout with edge bundling for clarity. Terms with a similarity score > 0.3 were linked by an edge (the thickness of the edge represents the similarity score). One term from each cluster was selected to have its term description shown in labels. The enrichment network visualization was shown with the intra-cluster and inter-cluster similarities of enriched terms. Cluster annotations are shown in color code. The Search Tool for the Retrieval of Interacting Genes/Proteins (STRING) was also applied to predict protein interactions (https://string-db.org/) A complex clustering network was generated by the 12 target gene sets.

### 4.3. Disease Analysis

To explore the disease relationships of the 12 interacting genes found among ivermectin, the 6 antioxidants, and COVID-19 in the CTD, two kinds of resources were utilized which informed the curation of chemical–disease and gene–disease relationships based on explorations in the literature and the adoption of gene–disease relationships from the database of the Online Mendelian Inheritance in Man (OMIM; McKusick-Nathans Institute of Genetic Medicine, Johns Hopkins University School of Medicine, Baltimore, MD, USA; http://www.ncbi.nlm.nih.gov/omim (accessed on 12 June 2022)). The OMIM is a freely available, comprehensive, and authoritative compendium of human genes and genetic phenotypes that is updated daily. Subsequently, potential human diseases were identified from the ivermectin and 6 antioxidants-gene-disease relationships inferred from the analyses of the CTD and STRING databases. The gene–disease network was visualized by the Gephi software (version 0.10.1; https://gephi.org/ (accessed on 27 August 2023)) [65].

### 4.4. Identifications of Target microRNA

MicroRNA (miRNA) has been found to act as a therapeutic option or biomarker to distinguish COVID-19 infection from other influenza diseases [50,66]. It has also been suggested that miRNA is the interplay between host and SARS-CoV-2, and that it could serve as the primary cause of SARS-CoV-2 accessing host cells [67]. Thus, we identified the potential targets of miRNA in patients treated with ivermectin and 6 antioxidants to provide more information on the relationship among ivermectin, the 6 antioxidants, and gene–disease relationship in pharmacogenomic analyses.

Targets of microRNA (miRNA) were predicted by applying the miRabel database, which has aggregated all human results based on four important miRNA target prediction algorithms (miRanda, PITA, SVmicrO, and TargetScan) (LITIS lab, University of Rouen Normandy, Mont-Saint-Aignan, France; http://bioinfo.univ-rouen.fr/mirabel/ (accessed on 12 June 2022)) [68]. These algorithms also specified additional characteristics for potential miRNA targets in the platform.

Potential target miRNAs of the 12 commonly curated genes were predicted by using the miRabel database [68]. For patients infected with COVID-19, the most up-regulated miRNA was hsa-miR-31-5p, whereas the most strongly down-regulated miRNA was has-miR-1275 [50]. In addition, two miRNAs were upregulatehashsa-miR-16-2-3P, hsa-miR-6501-5P) and two miRNAs were downregulated (hsa-miR-183-5p, hsa-miR-627-5p) [51]. The six miRNAs were further selected to analyze the target genes of the 12 commonly curated genes found among ivermectin, 6 antioxidants, and COVID-19.

## 5. Conclusions

Currently, no direct data concerning pharmacogenetics of ivermectin and the potential antioxidants in patients with COVID-19 are available. We have performed a computational analysis to establish the pharmacogenetic profile of the 12 common genes/proteins from ivermectin and the 6 investigated antioxidants by using the pharmacogenomic approach. This study elucidates molecular mechanisms and potential modes of action of the co-responses of the 12 common genes/proteins from ivermectin and the 6 investigated antioxidants to COVID-19. Diseases most associated with COVID-19 were found to be diabetes mellitus, followed by ischemia, and reperfusion injury. Furthermore, the information provided in this study could facilitate personalized medications through thw understanding of the safety, efficacy, and modes of action of repurposing drugs against COVID-19 in individuals with different gene profiles.

## Figures and Tables

**Table 1:** The 12 common curated genes.

Gene Symbols	Ensembl ID	Gene Names	Location
BAX	ENSG00000087088	BCL2 Associated X, Apoptosis Regulator	19q13.33
BCL2	ENSG00000171791	BCL2 Apoptosis Regulator	18q21.33
CASP3	ENSG00000164305	Caspase 3	4q35.1
CAT	ENSG00000121691	Catalase	11p13
CDH1	ENSG00000039068	Cadherin 1	16q22.1
CTNNB1	ENSG00000168036	Catenin Beta 1	3p22.1
GSR	ENSG00000104687	Glutathione-Disulfide Reductase	8p12
IL1B	ENSG00000125538	Interleukin 1 Beta	2q14.1
IL6	ENSG00000136244	Interleukin 6	7p15.3
NOS2	ENSG00000007171	Nitric Oxide Synthase 2	17q11.2
PARP1	ENSG00000143799	Poly(ADP-Ribose) Polymerase 1	1q42.12
TNF	ENSG00000232810	Tumor Necrosis Factor	6p21.33

**Table 2:** The potential target miRNAs of the 12 commonly curated genes were predicted using the miRabel database.

hsa-miR-16-2-3p								
Gene	miRabel Score	PITA	miRanda	SVMicrO	TargetScan	ExpVal	5′UTR	CDS
TNF	0.988286972	-	2668	15378	-	NO	NO	NO
CASP3	0.100207001	-	127	1966	105	NO	NO	YES
IL1B	0.996285021	-	4743	15,246	-	NO	NO	YES
BCL2	0.824105024	-	3263	10,901	1647	NO	NO	NO
IL6	0.752004027	-	1639	1959	-	NO	NO	NO
GSR	0.962409019	-	5136	8872	3521	NO	NO	NO
CTNNB1	0.994284987	-	4179	4578	-	NO	NO	NO
hsa-miR-183-5p								
Gene	miRabel Score	PITA	miRanda	SVMicrO	TargetScan	ExpVal	5′UTR	CDS
CASP3	0.983915985	2398	-	8157	-	NO	NO	NO
NOS2	0.994018972	4567	-	-	-	NO	NO	NO
BCL2	0.894342005	2504	5100	16,998	-	NO	NO	NO
IL6	0.994167984	-	4906	13,116	-	NO	NO	NO
GSR	0.879396021	4974	-	11,344	1877	YES	NO	YES
CTNNB1	0.984951973	-	2573	8458	-	NO	NO	NO
hsa-miR-6501-5p								
Gene	miRabel Score	PITA	miRanda	SVMicrO	TargetScan	ExpVal	5′UTR	CDS
TNF	0.989279985	-	-	-	1104	NO	NO	YES
CASP3	0.968086004	-	-	-	235	NO	NO	YES
BCL2	0.988623023	-	-	-	1060	NO	NO	YES
GSR	0.999303997	-	-	-	2847	YES	NO	YES
hsa-miR-627-5p								
Gene	miRabel Score	PITA	miRanda	SVMicrO	TargetScan	ExpVal	5′UTR	CDS
CASP3	0.961603999	3997	4606	4334	-	NO	NO	NO
IL1B	0.010888	3198	1449	550	509	NO	NO	YES
BCL2	0.526557028	2000	4630	2157	-	NO	NO	NO
IL6	0.970826983	-	853	14,610	-	NO	NO	NO
CTNNB1	0.016702199	1179	2532	1986	697	NO	NO	NO
hsa-miR-31-5p								
Gene	miRabel Score	PITA	miRanda	SVMicrO	TargetScan	ExpVal	5′UTR	CDS
BCL2	0.99229598	3762	-	-	-	NO	NO	NO
PARP1	0.211814001	3328	424	-	1367	YES	NO	YES
CTNNB1	0.72943902	2940	432	-	-	NO	NO	NO
hsa-miR-1275								
Gene	miRabel Score	PITA	miRanda	SVMicrO	TargetScan	ExpVal	5′UTR	CDS
CASP3	0.990505993	3998	-	5005	-	NO	YES	NO
NOS2	0.968831003	858	-	-	-	NO	NO	NO
BCL2	0.233618006	4952	6014	4709	2540	NO	NO	YES
PARP1	0.996828973	6499	-	10,247	-	NO	NO	YES
CTNNB1	0.996882021	-	6808	14,157	-	NO	YES	NO

## Data Availability

Data are provided in Supplementary Materials and are available on request.

## Supplementary Materials

The following supporting information can be downloaded at: https://www.mdpi.com/article/10.3390/ijms242015471/s1.
